# Effectiveness and safety of dupilumab in moderate‐to‐severe atopic dermatitis patients with chronic renal insufficiency: a real‐world retrospective study in China

**DOI:** 10.1002/mco2.707

**Published:** 2024-09-20

**Authors:** Cong Peng, Qiaozhi Cao, Feng Xiong, Hui Xu, Jie Li

**Affiliations:** ^1^ Department of dermatology, National Clinical Research Center for Geriatric Disorders Xiangya Hospital Central South University Changsha China; ^2^ The Division of of Nephrology Xiangya Hospital , Central South University Changsha China

Dear Editor,

Atopic dermatitis (AD) is a chronic, inflammatory, pruritic skin disorder with a complex etiology,^1^ characterized by a T helper 2 (Th2) immune response phenotype. The inflammatory factors, such as IL‐4, IL‐13, and IL‐31, produced by Th2 cells are significantly increased in AD patients.^2^ AD affects 3–10% of adults and 10–20% of children globally and is associated with substantial influence on their quality of life or families in severe cases.^1^, ^2^ Long‐term treatment options are limited for patients with moderate‐to‐severe AD.^1^ Dupilumab, a monoclonal antibody blocking signaling of IL‐4 and IL‐13, has been approved for the treatment of moderate‐to‐severe AD.^1^ Blocking IL‐4/13 is effective in reducing Th2 response. Dupilumab has exhibited favorable efficacy and safety for the treatment of AD in randomized, placebo‐controlled, double‐blind clinical trials, and real‐world studies.^1^, ^3^ However, data on the use of dupilumab in AD patients accompanied by chronic renal insufficiency (CRI) are limited. This study aimed to observe the effectiveness and safety of dupilumab in AD patients with CRI.

Eighteen moderate‐to‐severe AD patients with CRI at Xiangya Hospital, Central South University from April 2021 to November 2023 were retrospectively reviewed. AD was diagnosed in accordance with Hanifin and Rajka criteria by dermatologists, while CRI as well as the stage of chronic kidney disease (CKD) were diagnosed and classified by professional nephrologists. The study was approved by the Ethics Committee of Xiangya Hospital, Central South University (Ethics approval number: #202212806). Written informed consent was obtained from all participants. All patients received subcutaneously dupilumab 600 mg for the initial dose, followed by 300 mg every 2 weeks. There was no combination of other systemic therapies for AD, except one patient combined with oral tacrolimus and mycophenolate mofetil after kidney transplantation. Concomitant usage of low‐ to mid‐potency topical corticosteroids and/or topical calcineurin inhibitors were permitted. The demographic and clinical characteristics of the patients were recorded. Disease severity and quality of life scores, including the SCORing atopic dermatitis (SCORAD), eczema area and severity index (EASI), investigator's global assessment (IGA), peak pruritus numerical rating scale (PP‐NRS), dermatology life quality index (DLQI), and patient oriented eczema measure (POEM) scores, were assessed at baseline and 2, 4, 12, 16, 24, 52, and 104 weeks after the initial dose. The difference in disease severity scores and quality of life scores were compared by paired *t*‐test or Wilcoxon signed‐rank test at baseline and each time point after treatment. The primary endpoint was an improvement of at least 75% on the EASI (EASI‐75). The secondary end point was an IGA score of 0 or 1 or an improvement of at least 4 points in the PP‐NRS.

Demographic and clinical characteristics of 18 patients are listed in Table S1. 72.2% (13 out of 18) of the patients developed AD after CRI. Among eight hemodialysis patients, 87.5% (seven out of eight) developed AD after starting hemodialysis treatment, suggesting that the occurrence of AD in CRI patients may be related to CRI and hemodialysis, and the underlying reason needs further investigation. After dupilumab treatment at week 16, week 24, week 52 and week 104, the average scores of SCORAD (20.72, 19.88, 22.24, 20.24, respectively), EASI (5.86, 5.36, 4.98, 2.38, respectively), IGA (1.87, 1.80, 1.71, 1.40, respectively), PP‐NRS (2.87, 2.67, 3.64, 3.4, respectively), POEM (6.75, 6.07, 8.43, 7.60, respectively), and DLQI (4.31, 3.67, 4.64, 5.80, respectively) were significantly reduced from baseline (Table S1) (*p *< 0.05). In addition, the percentages of patients achieving EASI‐50, EASI‐75, EASI‐90, IGA0/1, PP‐NRS, and DLQI improvement ≥4 points were increased from baseline to week 104 (Figure 1).

**FIGURE 1:**
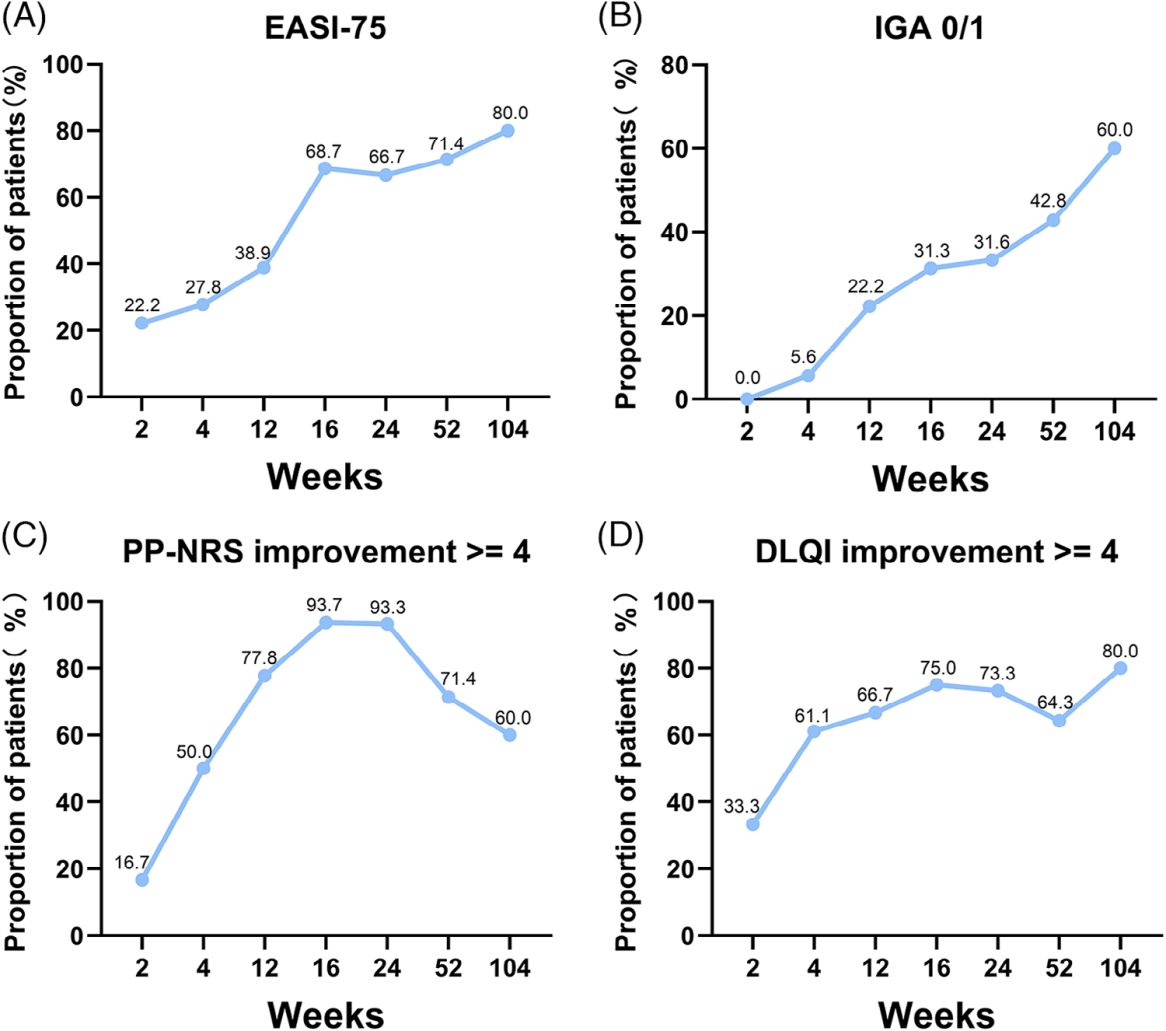
Percentages of patients who achieved (A) EASI‐75, (B) IGA0/1, (C) PP‐NRS, and (D) DLQI improvement ≥4 points at week 2 (*n* = 18), week 4 (*n* = 18), week 12 (*n* = 18), week 16 (*n* = 16), week 24 (*n* = 15), week 52 (*n* = 14), and week 104 (*n* = 5) after dupilumab treatment. EASI, eczema area and severity index; IGA, investigator's global assessment; PP‐NRS, peak pruritus numerical rating scale; DLQI, dermatology life quality index.

In our patients, percentages of achieving EASI‐75, EASI‐90 at week 16 are similar to those in a clinical trial^1^ excluding patients with CRI (68.7 vs. 69.0%, 31.2 vs. 40.0%, respectively), but the percentage of EASI‐90 at week 52 in our patients is lower than that in clinical trial data (35.7 vs. 51.0%). It may be partly due to the discontinuation of dupilumab in our four patients before week 52. Interestingly, percentage of PP‐NRS4 at week 16 in our patients (93.7%) is much higher than that reported in the clinical trial (59.0%).^1^ The discrepancy between our study and others may be due to the concomitant CRI, race, age, and limited sample size, etc., which suggests that dupilumab may be beneficial for uremic pruritus. Pruritus is a common symptom associated with CRI, which causes are still unclear. Serum interleukin (IL)‐31 is elevated in patients with uremic pruritus.^4^ In addition, IL‐31 is positively correlated with the intensity of uremic pruritus.^4^ It is possible that dupilumab indirectly reduces pruritus by decreasing production of IL‐31 by Th2 cells.^2^ In case reports, dupilumab can successfully treat patients with severe uremic pruritus.^5^ In our patients, disease severity and quality of life significantly improved after dupilumab treatment and sustained for 104 weeks in AD patients with different stage of CKD (Table S1). Moreover, during this long follow‐up period, dupilumab was well tolerated, neither adverse effects nor deterioration of renal function occurred.

This study has some limitations. First, this is a single‐center, hospital‐based study, which may have selective bias. Second, the sample size is limited.

This real‐world study presents the results of a cohort of Chinese AD patients with CRI who used dupilumab. In conclusion, our findings suggest that dupilumab was successful at improving signs and symptoms and was as an efficacious and safe treatment option for AD patients accompanied by CRI. Further study with multicenter and larger samples is warrant to provide more evidence for dupilumab in AD patients with CRI.

## AUTHOR CONTRIBUTIONS

Cong Peng, Qiaozhi Cao, and Feng Xiong wrote and revised the manuscript. Hui Xu diagnosed and classified CRI as well as the stage of CKD, revised the manuscript. Jie Li diagnosed and treat the patients, wrote and revised the manuscript. All authors have read and approved the final manuscript.

## CONFLICT OF INTEREST STATEMENT

The authors declare no conflicts of interest.

## ETHICS STATEMENT

This paper was reviewed and approved by the Ethic Committee of the Xiangya Hospital, Central South University; approval number #202212806. Written informed consent was obtained from all participants.

## Supporting information

Supporting Information

## Data Availability

The data that support the findings of this study are available from the corresponding author upon reasonable request.
