# Supplementary material for: Effectiveness and safety of dupilumab in moderate‐to‐severe atopic dermatitis patients with chronic renal insufficiency: a real‐world retrospective study in China
Source: MedComm (2020). 2024 Sep 20;5(10):e707. doi: 10.1002/mco2.707 (PMC11413493; doi:10.1002/mco2.707)
Supplement: Supplementary file 1 — Supporting Information [file MCO2-5-e707-s001.docx]

Effectiveness and safety of dupilumab in moderate-to-severe atopic dermatitis patients with chronic renal insufficiency: A real-world retrospective study in China

Authors: Cong Peng^1, 2#^, Qiaozhi Cao ^1, 2 #^, Feng Xiong^1, 2#^, Hui Xu **^1^**^,^ **^2^**^#^ , Jie Li ^✉1, 2^

Affiliations:

^1^ Department of dermatology, Xiangya Hospital, Central South University, Changsha, China.

^2^ The Division of Nephrology, Xiangya Hospital of the Central South University, Changsha, China.

Corresponding Authors: Jie Li, Department of Dermatology, Xiangya Hospital, Central South University, Changsha, Hunan, China. Fax: +86 073184327332. E-mail: xylijie@csu.edu.cn

#These authors contributed equally to this study.

**Materials and Methods**

**Study design and patients**

**Patients**

Inclusion criteria were: 1) resistant or unable to tolerate conventional treatments (such as topical and/or systemic corticosteroids, immunosuppressants); 2)those who were diagnosed chronic renal insufficiency by nephrologist; 3)those who were willing to receive dupilumab treatment; 4) signed informed consent forms. Exclusion criteria were: 1) active infection (including tuberculosis, HIV/AIDS, parasites or viral hepatitis); 2) allergic to dupilumab; 3) with other uncontrolled or serious systemic diseases, or malignancy, such as gammopathies, disorders with elevated peripheral blood eosinophils(e.g. eosinophilia), and any other serious blood dyscrasias; 4) pregnancy or lactation; 5) other conditions that need to be differentiated from AD such as bullous disease, scabies, psoriasis, insect bite, folliculitis, acne, habitual picking, lymphomatoid papulosis, dermatitis herpetiformis, chronic actinic dermatitis, sporotrichosis should be excluded by experienced doctors and examination such as biopsy, immunofluorescence.

**Measurements**

Disease severity scores were evaluated by SCORing Atopic Dermatitis (SCORAD), Eczema Area and Severity Index(EASI), Investigator's Global Assessment (IGA), Peak Pruritus Numerical Rating Scale(PP-NRS). Patients’ quality of life was evaluated by the Dermatology Life Quality Index(DLQI), and Patient Oriented Eczema Measure(POEM) scores. Stage of chronic kidney disease(CKD) was classified based on eGFR. The primary endpoint was an improvement of at least 75% on the EASI (EASI-75). The secondary end point was an IGA score of 0 or 1 or an improvement of at least 4 points in the PP-NRS.

**Data collection**

Demographic and clinical data of the patients were collected at baseline, week 2, 4, 12, 16, 24, 52 and week 104. Information including age, gender, disease duration, body mass index (BMI), disease severity scores, family history of atopy, allergic diseases concomitant, comorbidities, previous treatments, and combination treatments were recorded. Serum total IgE levels, Blood creatinine level, as well as counts of peripheral blood eosinophils at baseline. All reported adverse events (AE) were recorded during the visit.

**Statistical analysis**

All analyses were conducted using GraphPad Prism software version 9.0.1 and SPSS 26.0 statistical software. Continuous values were reported as mean and standard deviation(SD) or standard error of mean(SEM). The difference in disease severity scores and quality of life scores were compared by Paired t-test or Wilcoxon signed-rank test at baseline and each time point after treatment. Statistical significance was defined as P<0.05.

**Table S1. Baseline demographic and clinical characteristics of AD patients with** **chronic renal insufficiency treated with dupilumab**

| Features |  | N=18 |
| --- | --- | --- |
| Sex,male,n(%) |  | 15(83.33%) |
| Age(years),mean±SD(range) |  | 68.83±8.50(51-84) |
| BMI(kg/m2),mean±SD(range) |  | 21.14±3.19(15.64-30.25) |
| Duration of AD(months),mean±SD(range)  Duration of Chronic renal insufficiency(months),mean±SD(range) |  | 47.86±84.77(0.5-360)  58.50±73.31(1-240) |
| Disease severity, n (%) |  |  |
| 25< SCORAD <50 |  | 9(50.00%) |
| SCORAD＞50 |  | 9(50.00%) |
| Extrinsic status, n (%)† |  | 10(55.56%) |
| Family history of atopy, n (%) |  | 6(33.33%) |
| Atopic/allergic diseases concomitant, n (%) |  |  |
| Allergic rhinitis |  | 3(16.66%) |
| Asthma |  | 1(5.56%) |
| Previous medication for AD, n (%)‡ |  |  |
| Systemic antihistamines |  | 18(100%) |
| Systematic corticosteroids |  | 1(5.56%) |
| Immunosuppressive therapies (i.e., cyclosporine, methotrexate) |  | 2(11.11%) |
| Oral tripterygium wilfordii |  | 2(11.11%) |
| Traditional Chinese medicine |  | 5(27.78%) |
| TCS |  | 18(100.00%) |
| TCI |  | 1(5.56%) |
| Concomitant medications, n (%) |  |  |
| TCS |  | 18(100%) |
| TCI |  | 5(27.78%) |
| Total IgE level (IU/ml), mean±SD(range) |  | 641.17±1164.09(33.8-5125.8) |
| Eosinophil count (×10^9^/L), mean±SD(range) |  | 1.40±2.01(0-8.2) |
| Blood creatinine level (μmoI/L),mean±SD(range) |  | 420.01±281.41(116-1000) |
| Stage of CKD, n (%) **§** |  |  |
| CKD 3 |  | 5(27.78%) |
| CKD 4 |  | 5(27.78%) |
| CKD 5 |  | 8(44.44%) |
| IGA (mean±SD) ¶ | baseline | 3.27±0.57 |
| CKD3 | baseline | 3.80±0.44 |
|  | week 16 | 2.50±1.00 |
| CKD4 | baseline | 3.00±0.00 |
|  | week 16 | 1.75±0.50 |
|  | week 104 | 2.00 |
| CKD5 | baseline | 3.12±0.64 |
|  | week 16 | 1.62±1.18 |
|  | week 104 | 1.25±0.50 |
| SCORAD (mean±SD) ¶ | baseline | 52.54±14.09 |
| CKD3 | baseline | 63.60±9.53 |
|  | week 16 | 24.85±25.28 |
| CKD4 | baseline | 51.20±11.95 |
|  | week 16 | 17.77±4.85 |
|  | week 104 | 25.60 |
| CKD5 | baseline | 46.47±14.76 |
|  | week 16 | 20.12±19.05 |
|  | week 104 | 18.90±6.48 |
| EASI (mean±SD) ¶ | baseline | 22.11±6.94 |
| CKD3 | baseline | 29.78±3.64 |
|  | week 16 | 8.37±8.70 |
| CKD4 | baseline | 18.82±4.53 |
|  | week 16 | 3.75±1.43 |
|  | week 104 | 4.20 |
| CKD5 | baseline | 19.38±6.28 |
|  | week 16 | 5.65±8.47 |
|  | week 104 | 1.92±0.85 |
| DLQI (mean±SD) ¶ | baseline | 13.33±5.99 |
| CKD3 | baseline | 13.20±6.09 |
|  | week 16 | 5.50±3.51 |
| CKD4 | baseline | 13.60±5.94 |
|  | week 16 | 3.75±0.95 |
|  | week 104 | 4.00 |
| CKD5 | baseline | 13.25±6.75 |
|  | week 16 | 4.00±4.14 |
|  | week 104 | 6.25±4.64 |
| POEM (mean±SD) ¶ | baseline | 17.72±5.34 |
| CKD3 | baseline | 19.80±6.87 |
|  | week 16 | 9.75±9.42 |
| CKD4 | baseline | 16.00±2.12 |
|  | week 16 | 4.75±1.70 |
|  | week 104 | 5.00 |
| CKD5 | baseline | 17.50±5.87 |
|  | week 16 | 6.25±5.52 |
|  | week 104 | 8.25±6.18 |
| PP-NRS (mean±SD) ¶ | baseline | 8.55±1.19 |
| CKD3 | baseline | 9.20±0.83 |
|  | week 16 | 4.00±2.70 |
| CKD4 | baseline | 8.60±0.89 |
|  | week 16 | 3.00±0.81 |
|  | week 104 | 3.00 |
| CKD5 | baseline | 8.12±1.45 |
|  | week 16 | 2.25±1.48 |
|  | week 104 | 3.50±1.91 |

† Intrinsic AD was defined as total serum IgE≤200 kU/L and extrinsic AD was total serum IgE>200 kU/L;

‡ Previous systemic medication has been discontinued for at least 4 months.

**§** Stage of CKD was classified based on eGFR. The eGFR of all the patients is less than 45ml·min^-1^·(1.73m^2^)^-1^ , therefore, the stage of our patients are CKD 3 and above.

Abbreviations: BMI, body mass index; TCS , topical corticosteroids; TCI , topical calcineurin inhibitors;

¶ CKD3: baseline: n=5; week 16: n=4; CKD4: baseline: n=5; week 16: n=4; week 104: n=1; CKD5: baseline, week 16: n=8; week 104: n=4.
